# Patterns of changing pregnancy intentions among women living with HIV in Canada

**DOI:** 10.1186/s12905-021-01492-1

**Published:** 2021-10-06

**Authors:** Lashanda Skerritt, Angela Kaida, Nadia O’Brien, Ann N. Burchell, Gillian Bartlett, Édénia Savoie, Isabelle Boucoiran, Rebecca Gormley, Mary Kestler, Deborah Money, Mona Loutfy, Alexandra de Pokomandy

**Affiliations:** 1 Department of Family Medicine, McGill University, Montreal, Canada; 2 Faculty of Health Sciences, Simon Fraser University, Burnaby, Canada; 3 Centre de Recherche du Centre Hospitalier de l’Université de Montreal (CRCHUM), Montreal, Canada; 4 Department of Family and Community Medicine, Centre for Urban Health Solutions, Li Ka Shing Knowledge Institute, St. Michael’s Hospital, Unity Health Toronto, Toronto, Canada; 5 Department of Family and Community Medicine, University of Missouri, Columbia, USA; 6 Chronic Viral Illness Service, McGill University Health Centre, Glen Site 1001 Decarie Blvd., Rm D02.4110, Montreal, QC H4A 3J1 Canada; 7 Women and Children’s Infectious Diseases Center, Centre Hospitalier Universitaire Sainte-Justine, Montreal, Canada; 8 British Columbia Centre for Excellence in HIV/AIDS, Vancouver, Canada; 9 Oak Tree Clinic, BC Women’s Hospital and Health Centre, Vancouver, Canada; 10 Faculty of Medicine, University of British Colombia, Vancouver, Canada; 11 Women’s College Research Institute, Women’s College Hospital, Toronto, Canada

**Keywords:** HIV, Pregnancy, Family planning services, Preconception care, Women’s health

## Abstract

**Background:**

Women with an undetectable viral load can become pregnant and have children with no risk of HIV transmission to their sexual partners and low risk of transmission to their infants. Contemporary pregnancy intentions of women living with HIV in Canada are poorly understood, evidenced by high rates of unintended pregnancy and low uptake of contraceptives.

**Methods:**

We used longitudinal survey data from the Canadian HIV Women’s Sexual and Reproductive Health Cohort Study (CHIWOS) to measure and compare pregnancy intentions (Yes *vs* No *vs* Unsure) at baseline, 18-months and 36-months follow-up (from 2013 to 2018) among women living with HIV of reproductive age (16–49 years) and potential. We used Sankey diagrams to depict changes in pregnancy intentions over time and multivariable logistic regression to examine the relationship between pregnancy intention within 2 years and subsequent pregnancy.

**Results:**

At baseline, 41.9% (119/284) of women intended to become pregnant, 43.3% did not, and 14.8% were unsure. Across 36-months of follow-up, 41.9% (119/284) of women changed their pregnancy intentions, with 25% changing from intending to not intending to become pregnant and 13.1% vice versa. Pregnancy intentions were not strongly associated with subsequent pregnancy between baseline and 18-months (aOR 1.44; 95% CI 0.53, 3.72) or between 18 and 36-months (aOR 2.17; 95% CI 0.92, 5.13).

**Conclusions:**

Our findings underscore the need for healthcare providers to engage in ongoing discussions with women living with HIV to support their dynamic pregnancy intentions.

## Introduction

The reproductive landscape for women living with HIV has changed significantly [1, 2], such that it is now possible for women engaged in HIV care to become pregnant and have children with no risk of HIV transmission to their partners and an extremely low risk to future infants [3–5]. Medical advances have contributed to the increased incidence of pregnancy and childbirth among women living with HIV [6], driven mainly by unintended pregnancies [7, 8]. In a survey of women living with HIV in the United States who had recently given birth, about half reported that their pregnancy was mistimed, and half felt that before becoming pregnant, they did not want to have a baby [9]. Poor maternal and child health outcomes associated with unintended pregnancies [10] likely extend to women living with HIV and may be exacerbated by structural forms of oppression, including HIV stigma, a persistent and known barrier to healthcare access [11, 12].

Pregnancy intentions include intending to become pregnant, pregnancy spacing, avoiding pregnancy, or being unsure about pregnancy intention. Estimates of pregnancy intentions among women living with HIV in Canada are from cross-sectional studies prior to undetectable = untransmittable (U = U) messaging [13, 14]. Therefore, they do not capture contemporary pregnancy intentions or their dynamic nature. Although pregnancy planning clinical guidelines exist to support healthcare providers in counselling women living with HIV about family planning and safe conception, discussions about pregnancy intentions between women and their healthcare providers are not routine and can be stigmatizing [15, 16]. Among women living with HIV of reproductive age in Canada, 60% have never discussed their reproductive goals with a healthcare provider since being diagnosed with HIV [17]. Additionally, uptake of effective contraceptive methods among women living with HIV who report wanting to avoid pregnancy is low [18], and the range of contraceptive methods used is more narrow compared to HIV negative women [19], underscoring the need and opportunity to better understand and address the sexual and reproductive health needs of women living with HIV.

Demands for improving patient-provider communication through routine screening of pregnancy intentions are increasing [20–24]. Existing evidence and recommendations, however, are based on women’s pregnancy intentions at one point in time and do not capture the dynamic complexity of reproductive decision-making. Further, few studies have investigated whether pregnancy intention predicts future pregnancy outcomes. The objectives of this study were to measure and compare the pregnancy intentions of women living with HIV in Canada over time and investigate the relationship between pregnancy intention within 2 years and subsequent pregnancy.

## Methods

### Study design and setting

We used longitudinal survey data from the Canadian HIV Women’s Sexual and Reproductive Health Cohort Study (CHIWOS), a longitudinal community-based participatory study [25] conducted by, for, and with women living with HIV in British Columbia, Ontario, and Quebec, where over 80% of women living with HIV in Canada reside [26]. In Canada, women living with HIV represent approximately 23% of people living with HIV [26]. HIV prevalence and incidence are higher among marginalized women, including women in poverty, women of Indigenous ancestry, women who identify as African, Caribbean, or Black, refugees and new immigrants, and sexual and gender minorities. For many women living with HIV, several of these marginalized identities intersect [27].

### Participants

Between August 2013 and May 2015, 1,422 women enrolled in CHIWOS and completed the baseline survey. CHIWOS eligibility criteria included self-identifying as a woman (including cis, trans, two-spirit, gender-queer, or questioning people who identified as women), 16 years of age or older, diagnosed with HIV, and living in one of the study provinces. Women were recruited using a non-random, purposive sampling approach [28, 29]. Participants completed a computer-based survey administered by Peer Research Associates (women living with HIV who completed research training) [25]. Two follow-up surveys were administered 18 and 36 months after the baseline visit. Baseline data were collected between 2015 and 2016, 18-month follow-up data between 2016 and 2017, and 36-month follow-up data between 2017 and 2018. Total study retention over 36-months was 66%.

For this analysis, we excluded women who were 50 years of age or older at baseline or unable to become pregnant (self-reported infertility, menopausal, post-menopausal, male sex assigned at birth). We also excluded women with missing data on pregnancy intentions across the three follow-up visits, either because they preferred not to answer the pregnancy intention question or were lost to follow-up.

All participants provided written or verbal informed consent. Ethical approval was granted from all participating institutional Research Ethics Boards, including Simon Fraser University, University of British Columbia/Providence Health Centre, Women’s College Hospital and McGill University Health Centre, and participating clinics and AIDS Service Organizations where requested.

### Measures

Pregnancy intention was measured by the baseline, 18-month, and 36-month follow-up survey question “Do you intend to become pregnant in the future?”, with responses categorized as “Yes,” “No,” or “Unsure.” We also measured pregnancy intention within 2 years by asking women who responded “Yes” to the former question, “When in the future do you intend to become pregnant?”. We then derived the following categories: “Intends to become pregnant within 2 years,” “Does not intend to become pregnant within 2 years,” and “Unsure.”

At each follow-up visit, women were asked how many pregnancies they had since their last study visit (including currently being pregnant). Participants who reported being pregnant at least once since their last study visit were assessed as having a pregnancy.

When assessing the relationship between reported pregnancy intention at baseline and 18-months (exposure) and subsequent pregnancies reported at 18 and 36-months (outcome), we considered as confounders women’s age, ethnicity, number of children, relationship status, educational attainment, and household income, as previous studies have identified these factors as strong determinants of both pregnancy intention and pregnancy outcomes [10]. The exposure and confounders were measured at the same time-points. Women who preferred not to answer the question about their relationship status (n = 1) were categorized as ‘single/ other’. We assumed that women who preferred not to answer the question about education (n = 1) had not completed high school.

### Statistical methods

Descriptive statistics were used to characterize the sample at baseline and to examine pregnancy intentions and subsequent pregnancy. Sankey diagrams [30] were used to depict longitudinal absolute (*n*) and relative frequencies (%) of pregnancy intentions at the three study visits. Sankey diagrams were also stratified by age category to account for differences in pregnancy intention between younger and older women.

Two separate multivariable logistic regression models were fit to investigate the relationship between pregnancy intention within 2 years and subsequent pregnancy between the baseline and 18-month visit (model 1) and between the 18-month and 36-month visit (model 2) while adjusting for potential confounders. Following recent calls to move away from reliance on statistical significance in interpreting research results [31], we adopted an approach to estimating proportions and measures of association, recognizing that *p* values should not drive the interpretation of statistical analyses. Based on previous literature [10, 13, 14, 32–35] and the expertise of clinicians and women living with HIV, we considered the following as potential confounders in our model: women’s age, race/ethnicity, number of children, relationship status, education, and household income.

A sub-analysis was performed to compare reported live births and pregnancy terminations over the 36-month study period among women 35 years of age and younger to women over 36 years of age using descriptive statistics. We also compared live births and pregnancy terminations across baseline pregnancy intention. All analyses were performed using R: A Language and Environment for Statistical Computing (R Foundation for Statistical Computing, Vienna, Austria, 2019).

## Results

**Fig. 1:**
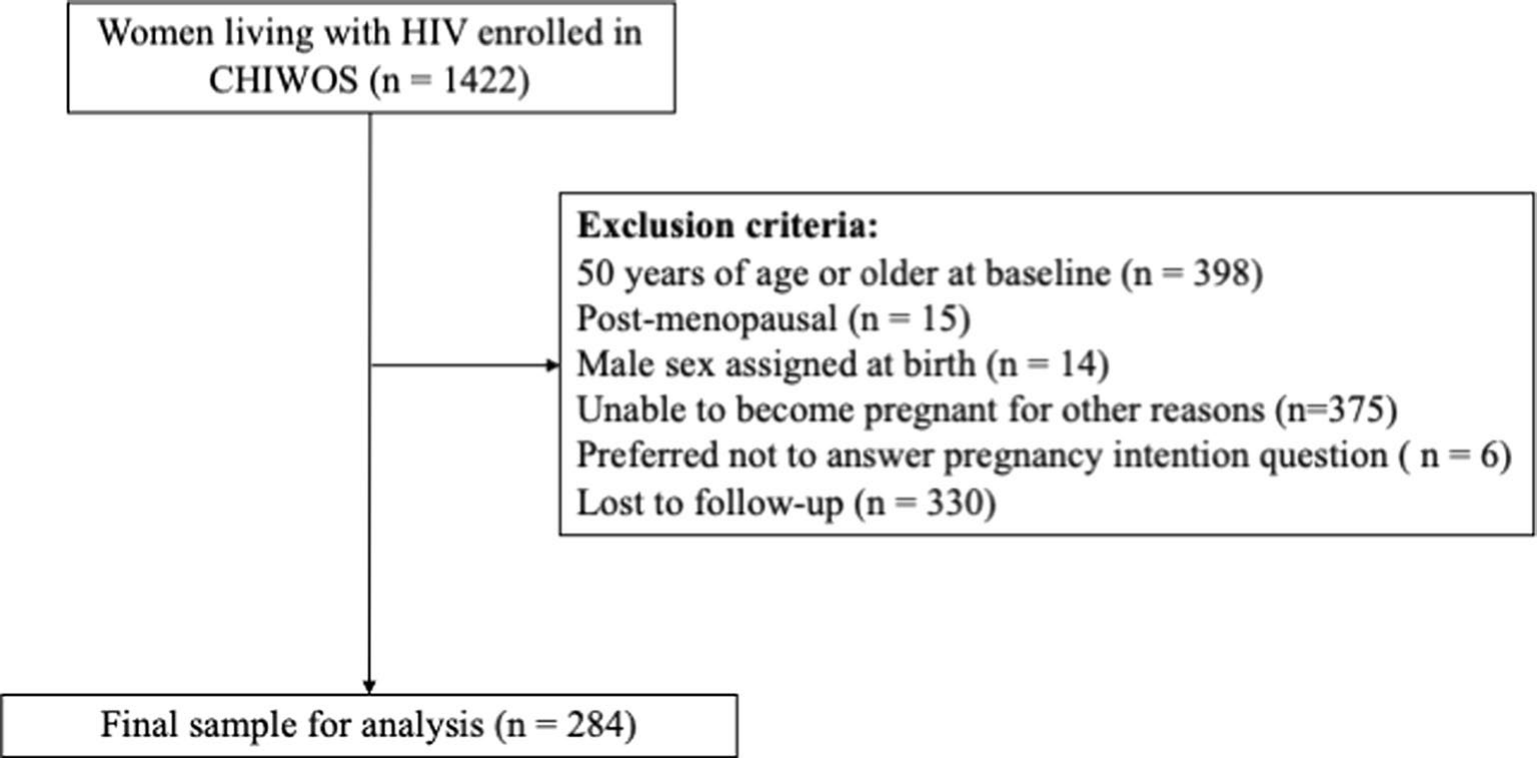
CHIWOS participant inclusion and exclusion criteria for analysis of pregnancy intentions

Of the 1422 women living with HIV enrolled in CHIWOS, 284 were included in this analysis (20.0% of the total cohort). We excluded 398 participants who were 50 years of age or older, 15 who were postmenopausal, 14 who were assigned male sex at birth, 375 who reported being unable to become pregnant for other reasons, 6 who preferred not to answer questions on pregnancy intention, and 330 who were lost to follow-up (Fig. 1).

### Baseline characteristics of the study sample

At baseline, the median age was 36.0 years [interquartile range 31.0–40.0]. Women living in Ontario represented the largest proportion (46.1%), followed by Quebec (31.0%) and British Columbia (22.9%). The majority of women identified as African/Caribbean/Black (54.2%), had a high school education or higher (83.1%), at least 1 child (67.3%), an annual household income of less than CAD 20,000 (52.1%), identified as heterosexual (86.6%), and were currently on ARTs (85.2%) (Table 1).

**Table 1:** Baseline demographic characteristics of participants in the Canadian HIV Women’s Sexual and Reproductive Health Cohort Study (CHIWOS) included in these analyses (n = 284)

	Overall (N = 284)
*Age (years)*	
Median [Q1, Q3]	36.0 [31.0, 40.0]
*Ethnicity*	
Indigenous	32 (11.3%)
African/Caribbean/Black	154 (54.2%)
White	79 (27.8%)
Other/Mixed	19 (6.7%)
*Province*	
British Columbia	65 (22.9%)
Ontario	131 (46.1%)
Quebec	88 (31.0%)
*Education attainment*	
Lower than high school	47 (16.5%)
High school or higher	236 (83.1%)
DK/PNTA	1 (0.4%)
*Relationship status*	
Married/Relationship/Common-law	117 (41.2%)
Single/Other/PNTA	131 (46.1%)
Separated/Divorced/Widowed	36 (12.7%)
*Number of children*	
None	93 (32.7%)
1 or 2	126 (44.4%)
3 or more	65 (22.9%)
*Household income (CAD)*	
< 20 K	148 (52.1%)
20 K-40 K	66 (23.2%)
> = 40 K	56 (19.7%)
DK/PNTA	14 (4.9%)
*ART use*	
Not currently but previously on ARTs	22 (7.7%)
Currently on ARTs	242 (85.2%)
Never on ARTs	19 (6.7%)
DK/PNTA	1 (0.4%)
*Sexual orientation*	
Heterosexual	246 (86.6%)
LGBTTQ	36 (12.7%)
DK/PNTA	2 (0.7%)

[Q1, Q3] first quartile, third quartile; DK/PNTA, don’t know or prefer not to answer; CAD, Canadian Dollars;

ART, antiretroviral therapy; LGBTTQ, Lesbian, Gay, Bisexual, Transgender, Two-Spirit and Queer

### Pregnancy intentions and patterns of changing intentions

**Fig. 2:**
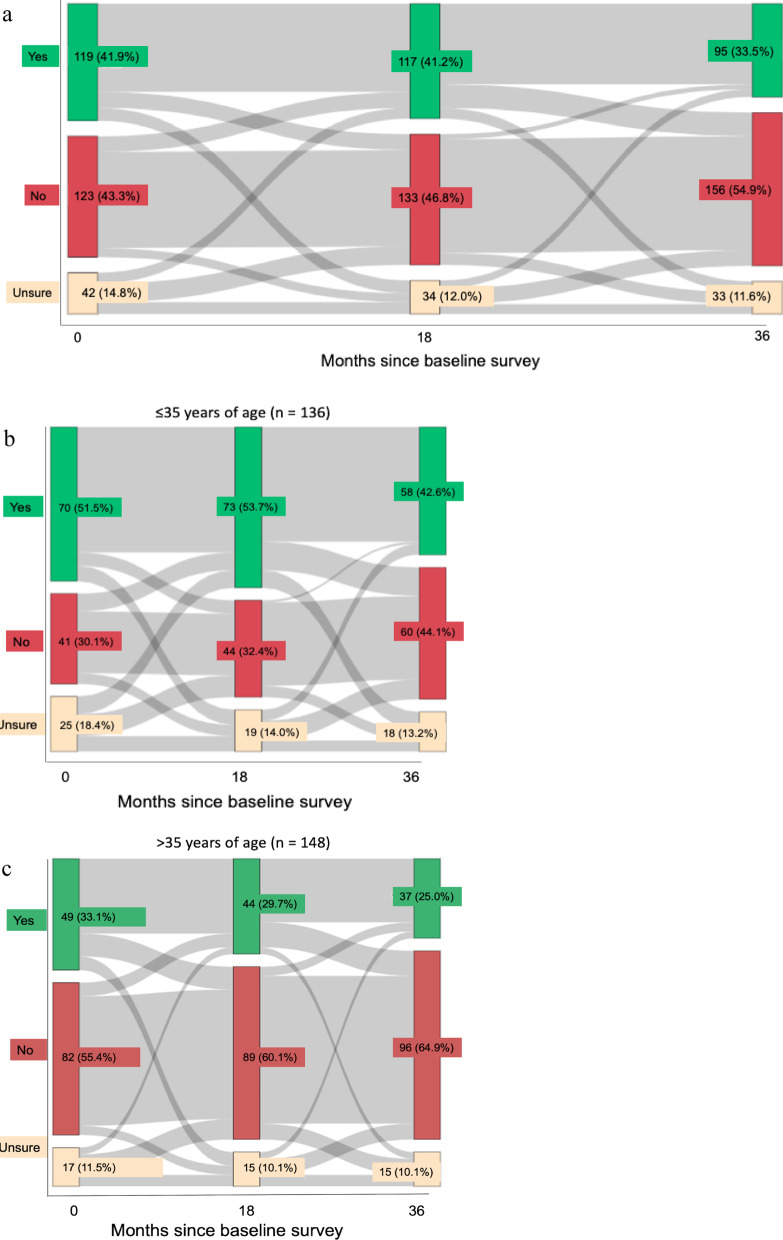
**a** Sankey diagram showing the proportion of participants who reported that they intended (green), did not intend (red) or who were unsure (beige) about whether to become pregnant in the future. The grey bars depict shifts in intention between surveys. The height of the grey bars is proportional to the number of participants. **b** Sankey diagram for women 35 years of age and younger (16–35 years of age). **c** Sankey diagram for women over 35 years of age (36–49 years of age)

At baseline, 41.9% (119/284) intended to become pregnant in the future, 43.3% (123/284) of women reported that they did not intend to become pregnant in the future, and 14.8% (42/284) were unsure. At 18-months, 41.2% (117/284) intended to become pregnant in the future, 46.8% (133/284) of women did not intend to become pregnant in the future, and 12.0% (34/284) were unsure. At 36-months, 33.5% (95/284) intended to become pregnant in the future, 54.9% (156/284) did not intend to become pregnant in the future, and 11.6% (33/284) were unsure. Figure 2a depicts reported pregnancy intentions and changes between the baseline, 18-month, and 36-month surveys among all participants included in the analysis.

Over the 36-month observation period, 58.1% (165/284) of women had no change in their pregnancy intentions. Among those who reported consistent pregnancy intentions, 86 (30.3% of total) intended to become pregnant throughout the study period, 74 (26.1% of total) did not, and 5 (1.8% of total) were unsure at each visit. Across 36 months, 41.9% (119/284) of women changed their pregnancy intentions, with 25% of changes from intending to not intending to become pregnant, and 13.1% from not intending to intending to become pregnant. Between baseline and 18-months and between 18 and 36-months, 29.6% and 26.8% of women changed their pregnancy intentions, respectively. Between baseline and 18-months, changes from being unsure to not intending to become pregnant accounted for 22.6% (19/84), the largest proportion, of changes in intention. Between 18 and 36-months, changes from intending to not intending to become pregnant accounted for 31.6% (24/76) of all observed changes. Among women 35 years of age and younger, 46.3% (63/136) changed their pregnancy intention over 36-months compared to 37.8% (56/148) among women over 35 (Fig. 2b, c).

### Pregnancy intention and subsequent pregnancy

We assessed the relationship between women’s pregnancy intentions within 2 years and pregnancies in the subsequent 18-months. Intention to become pregnant within 2 years was 23.3% (58/284) at baseline (Table 2) and 27.1% (77/284) at 18-months (Table 3). Between baseline and 18-months, 15.5% (9/58) of women who intended to become pregnant within 2 years did so, 12.5% (23/184) of women who did not intend to become pregnant became pregnant, and 14.3% (6/42) who were unsure became pregnant (Table 2). Between 18 and 36-months, 20.8% (16/77) of women who intended to give birth within 2 years did so, 11.0% (19/173) of women who did not intend to become pregnant had a pregnancy, and 5.9% (2/34) who were unsure became pregnant (Table 3).

**Table 2:** Association between baseline pregnancy intention within 2 years and pregnancy in the subsequent 18-months

Pregnancy intention	n (%) became pregnant in subsequent 18-months	OR (95%CI)	Adjusted^a^ OR (95%CI)
Intends to become pregnant within 2 years (n = 58)	9 (15.5%)	1.29 (0.53, 2.88)	1.44 (0.53, 3.72)
Does not intend to become pregnant within 2 years (n = 184)	23 (12.5%)	Ref	Ref
Unsure (n = 42)	6 (14.3%)	1.17 (0.41, 2.92)	1.10 (0.37, 2.92)

^a^Adjusted for age category, ethnicity, number of children, relationship status at time of survey, education, and household income

**Table 3:** Association between 18-month pregnancy intention within 2 years and pregnancy in the subsequent 18-months

Pregnancy intention	n (%) became pregnant in subsequent 18-months	OR (95%CI)	Adjusted^a^ OR (95%CI)
Intends to become pregnant within 2 years (n = 77)	16 (20.8%)	2.13 (1.02, 4.41)	2.17 (0.92, 5.13)
Does not intend to become pregnant within 2 years (n = 173)	19 (11.0%)	Ref	Ref
Unsure (n = 34)	2 (5.9%)	0.51 (0.08, 1.87)	0.26 (0.04, 1.14)

^a^Adjusted for age category, ethnicity, number of children, relationship status at time of survey, education, and household income

After adjusting for potential confounders, there was no association observed between reporting an intention to become pregnant within 2 years at baseline and pregnancy in the subsequent 18-months (adjusted OR 1.44; 95% confidence interval 0.53, 3.72) or between being unsure at baseline and pregnancy by 18-months (aOR 1.10; 95% CI 0.37, 2.92) (Table 2). Intending to become pregnant within the next 2 years at the 18-month follow-up study visit was associated with 2.17 times higher adjusted odds of pregnancy by 36-months (95% CI 0.92, 5.13). Being unsure about pregnancy intention was associated with lower odds of subsequent pregnancy (aOR 0.26; 95% CI 0.04, 1.14) (Table 3). These relationships, however, were not statistically significant.

### Sub-analysis: pregnancy outcomes

**Fig. 3:**
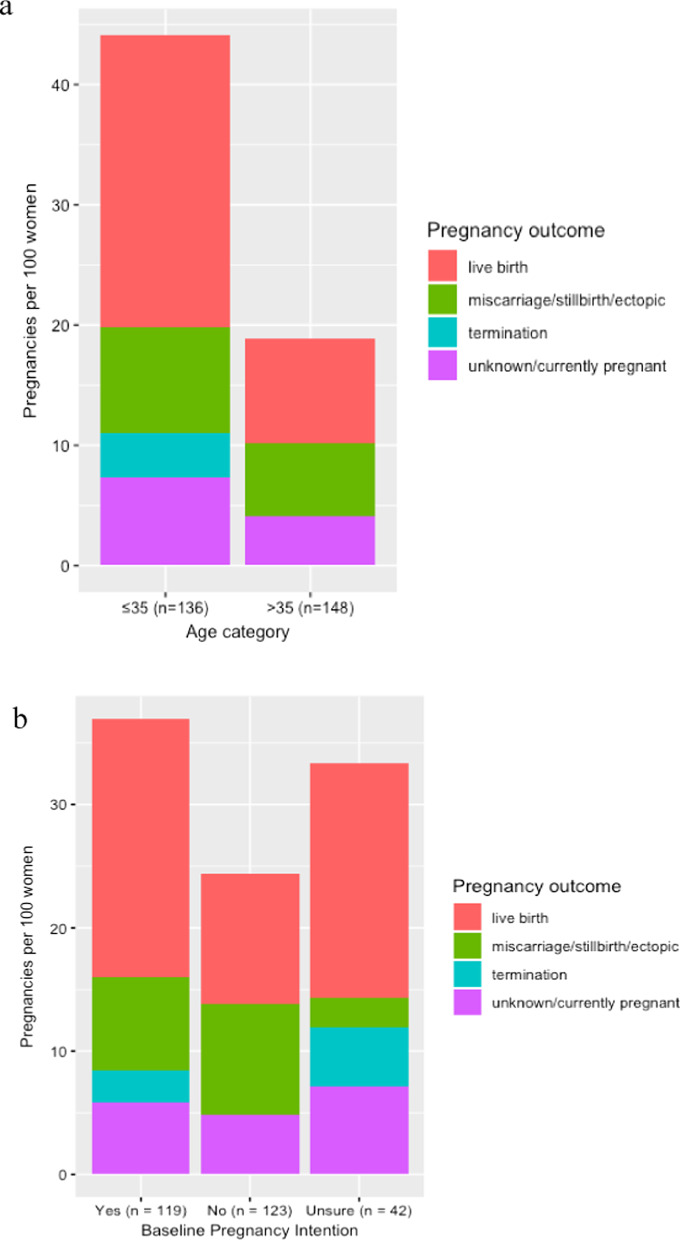
**a** Pregnancy outcomes per 100 women over 36-month by age category. **b** Pregnancy outcomes per 100 women over 36-months by baseline pregnancy intention

Figure 3 shows pregnancy outcomes over 36-months per 100 women by age category and baseline pregnancy intention. Among the 136 women aged 16–35 in our study, there were 60 pregnancies during the study period. Of those pregnancies, 33 ended in live births, and 5 were terminated. Among the 148 women aged 36–49, there were 28 pregnancies. Of those, 13 ended in live births, and none were terminated (Fig. 3a). There were 44 pregnancies reported among the 119 women who intended to become pregnant at baseline, of which 25 ended in live births, and 3 were terminated. There were 30 pregnancies among the 123 women who did not intend to become pregnant at baseline, of which 13 ended in live births, and none were terminated. Among the 42 women who were unsure about their pregnancy intention, 14 became pregnant, 8 had live births and 2 pregnancies were terminated (Fig. 3b).

## Discussion

Among this sample of women aged 16–49 years and of reproductive potential living with HIV in Canada, we observed diverse and dynamic pregnancy intentions over a 36-month follow-up period. Over one-quarter of women changed their pregnancy intention over 18-months, and 42% did so over 36 months. At baseline, 43% of women living with HIV did not intend to become pregnant in the future; however, only 26% reported not intending to become pregnant at all 3 study visits. Subsequent pregnancies were not strongly associated with initial pregnancy intentions, indicating the dynamic nature of pregnancy intention and underscoring the need for ongoing reproductive discussions between women and their healthcare providers.

Our finding that 43% of women intended to become pregnant at baseline is similar to that reported in a meta-analysis estimating that 42% of women living with HIV in high-income countries between 1997 and 2015 intended to become pregnant in the future [32]. The proportion was lower than that reported in a cross-sectional study of slightly older and mostly immigrant women living with HIV in Ontario between 2007 and 2009 [14], where 57% intended to give birth in the future. The proportion was higher than the 39% of women living with HIV in British Columbia surveyed in 2007 [13] who intended to give birth in the future. The study included a larger percentage of Indigenous women and single women compared to our analyses. These studies reported pregnancy intentions before the era of U = U and were cross-sectional.

Our longitudinal study revealed that intentions were dynamic, changing significantly over 36-months. The proportion of women not desiring pregnancy increased from 40% at baseline to 55% at the 36-month visit. Close to half of all observed shifts in pregnancy intentions throughout the study were changes from intending pregnancy or being unsure to not wanting to become pregnant. These results may be explained by increasing age [14] or pregnancies that occurred over that time, but also demonstrate that for many women living with HIV, reproductive health needs change over a few years. Only one-quarter of women in the study consistently reported not intending to become pregnant at each study visit. At baseline, around 20% of women were unsure whether they wanted to become pregnant in the future. A study published in 2013 of men and women living with HIV in Los Angeles found that 13% of study participants who responded “no” to the survey question “do you wish to have a/another child?”, also responded “yes” when asked “Would your desire to have a/another child change if you knew you could have a child with limited risk of transmitting HIV to your partner and the child” [36]. These results may reflect feeling inadequately informed to make pregnancy decisions given the changing reproductive landscape, particularly for women living with HIV [37–42].

Previous research has described the factors that shape pregnancy desires and intentions [13, 14, 32, 33]. Our results suggest that pregnancy intention at one point in time are not strongly associated with future pregnancy occurrence. Although intending to become pregnant in the future was positively associated with subsequent pregnancy, the observed relationship was not statistically significant. The weak observed relationship at baseline and 18-months may be explained by social desirability bias due to stigma related to HIV and motherhood [34, 43] but could also be explained by the larger social context of women’s lives and factors that influence women’s choice or lack of choice in becoming pregnant or avoiding pregnancy, such as relationships, health, employment, income and housing security [44]. Moreover, pregnancy terminations were more common among women who intended to become pregnant at baseline compared to women who did not, further demonstrating that the relationship between pregnancy intention and outcome is complex. A study of the sexual and intimate relationship experiences of women living with HIV in Canada found that women in long-term/unhappy sexual relationships or short-term sexual relationships were more likely to experience low levels of power equity [45]. Women in these relationships may not feel empowered to decide whether and when to have children. Low uptake of long-acting and preferred contraceptive methods among women living with HIV [19] may also explain the weak association between pregnancy intentions and outcomes. Unintended pregnancies are associated with an increased likelihood of negative feelings and experiences during pregnancy and in the postpartum period [46]. On the other hand, women living with HIV describe the fear and criminalization of HIV transmission to be a barrier to engaging in sexual relationships [47, 48], despite the emergence of evidence showing people living with HIV who are taking ART and have a suppressed viral load have effectively no risk of transmitting HIV to their sexual partners [1]. Women’s pregnancy decisions occur within complex social contexts shaped by intimate relationship power inequity, economic precarity, HIV-related stigma, and HIV criminalization laws that all compete with their desires to avoid pregnancy or become pregnant.

Healthcare providers should be aware of the social and structural factors that influence the relationship between pregnancy intention and pregnancy outcome. Despite the large proportion of women whose intentions changed over the study, analyses of data from the same cohort found that reproductive discussions are not routine and account for the largest measured gap in comprehensive healthcare for women living with HIV [17, 49]. Supporting the uptake of effective contraception and clinical follow-up is particularly important to help women living with HIV prevent unwanted and unplanned pregnancies [35] and improve uptake of long-acting and hormonal contraceptives, which has been observed to be lower among women living with HIV in Canada compared to the general population [19]. Women living with HIV have described the support and counselling received by their healthcare providers as instrumental to their decision-making [35, 44]. However, most women living with HIV receive care from HIV specialized settings where reproductive discussions are less likely to occur compared to non-HIV specialized settings [17]. According to primary care providers, competing health priorities are the main barrier to asking women about their pregnancy intentions [50]. Strategies for promoting these discussions include delegating from physicians to members of multidisciplinary care teams [51, 52], raising the topic of pregnancy at one visit and following up on at a subsequent visit [50], using waiting room tools to support patient agency initiating reproductive discussions, introducing comprehensive training on pregnancy intention discussions and periodic check-ins between healthcare providers in the same clinics to facilitate sharing of strategies and best practices [50]. Community and AIDS service organizations can also support these conversations and services by promoting empowerment and information about initiating reproductive discussions with healthcare providers.

### Limitations and strengths

This study has limitations. We excluded women living with HIV who were missing longitudinal data on their reproductive intentions. Women lost to follow-up were less likely to be engaged in HIV care and more likely to have a detectable viral load (data not shown). According to previous studies, this population is less likely to desire to have children [33]. Moreover, persistent HIV-related stigma associated with pregnancy and motherhood may have led some women to report not intending to become pregnant or being unsure rather than reporting that they intend to become pregnant in the future. On the other hand, women living with HIV who desire to become pregnant in the future may be more likely to participate in research focused on Sexual and Reproductive Health among women living with HIV, which may have led to an overestimate of the proportion of women living with HIV who intend to become pregnant in the future. Our study was powered to detect only large associations between pregnancy intention and subsequent pregnancy. The small sample size should be considered in the interpretation of the estimated associations. Although these associations were not found to be statistically significant in this analysis, the direction of the association was positive and should be further investigated in future studies with larger sample sizes. Residual confounding and misclassification of covariates could have resulted in underestimating or overestimating the true effect of pregnancy intention on subsequent pregnancy because of incomplete adjustment [53]. This study was conducted in the context of universal healthcare coverage and in the global North. The findings may not extend to other contexts where financial and medical barriers may have a greater impact on pregnancy intentions and subsequent pregnancies.

This study has several strengths. Surveys were administered by PRAs, which may have made participants more comfortable answering sensitive questions. Recruitment strategies extended beyond clinics and aimed to include women less engaged in healthcare and research. Our prospective assessment of pregnancy intention overcomes biases inherent in previous studies that collected pregnancy intention data retrospectively [7, 32, 54, 55].

The findings from this study have important implications for family planning counselling. To support the contraceptive and pregnancy planning needs of women living with HIV, healthcare providers should not only ask women about their pregnancy intentions but should also aim to create non-stigmatizing, trauma-aware, and women-centred environments [52, 56], normalizing these discussions. Discussing reproductive goals once with women living with HIV is not sufficient to address changing pregnancy intentions. Nor is it sufficient to ask about pregnancy intentions at a pre-specified frequency. Rather than approaching conversations around pregnancy and family planning as a routine screening question, these discussions need to be left open so that women living with HIV can discuss their intentions as they evolve and receive the counselling that aligns with their evolving needs and considers the social contexts and power dynamics that influence their reproductive decision-making [56, 57].

## Conclusion

This study demonstrates that women living with HIV have a diverse range of pregnancy intentions that change over time. It provides a crucial understanding of both the dynamic property of pregnancy intentions and the social contexts that influence the relationship between women’s intentions and their reality. Healthcare providers should promote safe and non-judgemental spaces where women feel comfortable discussing their reproductive intentions as they evolve. Promoting open and ongoing discussions is needed to provide women living with HIV with the support and counselling they need.

## Data Availability

The datasets used and/or analysed during the current study are available from the corresponding author on reasonable request.
